# Impact of central antagonist of cholecystokinin-1 receptors GB-115 on cognitive functions in patients with Generalized Anxiety Disorder

**DOI:** 10.1192/j.eurpsy.2022.490

**Published:** 2022-09-01

**Authors:** O. Dorofeeva, M. Metlina, A. Chepeliuk, M. Vinogradova

**Affiliations:** 1 FSBI “Zakusov Institute Of Pharmacology”, Department Of Pharmacological Genetics, Moscow, Russian Federation; 2 Lomonosov Moscow State University, Department Of Neuro- And Pathopsychology, Faculty Of Psychology, Moscow, Russian Federation

**Keywords:** anxiety disorder, cholecystokinin, anxiolytic, cognitive functions

## Abstract

**Introduction:**

Generalized anxiety disorder (GAD) is associated with reduced attention, inhibition, decrease of processing speed. The impact of a new peptide antagonist of central cholecystokinin-1 receptors (GB-115) on cognitive processes in patients with GAD is reported.

**Objectives:**

To research the cognitive effects of GB-115 in patients with GAD.

**Methods:**

25 patients with GAD in ICD-10 (mean age 35,76±8,55 years) treated with GB-115 in clinically relevant dose (6 mg/d) were enrolled to the study. The evaluation of cognitive functions was conducted at background, Day 3, Day 7, Day 14 and Day 21. The laboratory test toolkit included reaction time test, Shulte-Platonov tables, attention tests (using hardware and software complex “NeuroSoft-PsychoTest”). Statistical significance was ascertained by Wilcoxon signed-rank test.

**Results:**

Speed of reaction time increased on the Day 7 (418,17±61,49 msec, p≤0,01), the Day 14 (422,25±70,69 msec, p≤0,01) and the Day 21 of treatment (406,5±52,79 msec, p≤0,01) in comparison with background (449,19±64,91). Attention parameters improved on the Day 3 (305,95±45,31 msec, p≤0,05) and the Day 21 of treatment (300,14±47,74 msec, p≤0,05) in comparison with the background (316,41±42,35 msec). Decrease of time in performance of tables of Shulte-Platonov was also observed on the Day 7 (59,40±13,71 sec, p≤0,01), the Day 14 (57,88±12,82 sec, p≤0,01) and the Day 21 (53,40±13,19 sec, p≤0,01) in comparison with the background (68,84±16,78 sec).

**Conclusions:**

GB-115 revealed cognitive effects such as an increase of processing speed and improvement of different aspects of attention (attentional resource allocation, attention span and switching) after the Day 7 of treatment.

**Disclosure:**

No significant relationships.

